# Genome-wide analysis of hepatic DNA methylation reveals impact of epigenetic aging on xenobiotic metabolism and transport genes in an aged mouse model

**DOI:** 10.1007/s11357-024-01137-9

**Published:** 2024-04-01

**Authors:** Sara Abudahab, Mohamad M. Kronfol, Mikhail G. Dozmorov, Thomas Campbell, Fay M. Jahr, Jasmine Nguyen, Ola AlAzzeh, Dalia Y. Al Saeedy, Ashley Victor, Sera Lee, Shravani Malay, Dana M. Lapato, Matthew S. Halquist, MaryPeace McRae, Laxmikant S. Deshpande, Patricia W. Slattum, Elvin T. Price, Joseph L. McClay

**Affiliations:** 1 Department of Pharmacotherapy and Outcomes Science, School of Pharmacy, Virginia Commonwealth University, Dr. Joseph L. McClay, 6Th floor Smith Building, 410 North 12Th Street, Medical College of Virginia Campus, Richmond, VA 23298-0533 USA; 2 Department of Biostatistics, Virginia Commonwealth University, Richmond, VA USA; 3 Department of Pathology, Virginia Commonwealth University, Richmond, VA USA; 4 Department of Pharmaceutics, Virginia Commonwealth University, Richmond, VA USA; 5 Department of Human and Molecular Genetics, Virginia Commonwealth University, Richmond, VA USA; 6 Department of Neurology, Virginia Commonwealth University, Richmond, VA USA; 7 Department of Pharmacology and Toxicology, Virginia Commonwealth University, Richmond, VA USA; 8 Virginia Center On Aging, Virginia Commonwealth University, Richmond, VA USA

**Keywords:** Aging, Hepatic xenobiotic metabolism, Hepatic transporters, Epigenetics, Cytochrome P450s

## Abstract

Hepatic xenobiotic metabolism and transport decline with age, while intact xenobiotic metabolism is associated with longevity. However, few studies have examined the genome-wide impact of epigenetic aging on these processes. We used reduced representation bisulfite sequencing (RRBS) to map DNA methylation changes in liver DNA from mice ages 4 and 24 months. We identified several thousand age-associated differentially methylated sites (a-DMS), many of which overlapped genes encoding Phase I and Phase II drug metabolizing enzymes, in addition to ABC and SLC classes of transporters. Notable genes harboring a-DMS were *Cyp1a2*, *Cyp2d9*, and *Abcc2* that encode orthologs of the human drug metabolizing enzymes CYP1A2 and CYP2D6, and the multidrug resistance protein 2 (MRP2) transporter. *Cyp2d9* hypermethylation with age was significantly associated with reduced gene expression, while *Abcc2* expression was unchanged with age. *Cyp1a2* lost methylation with age while, counterintuitively, its expression also reduced with age. We hypothesized that age-related dysregulation of the hepatic transcriptional machinery caused down-regulation of genes despite age-related hypomethylation. Bioinformatic analysis of hypomethylated a-DMS in our sample found them to be highly enriched for hepatic nuclear factor 4 alpha (HNF4α) binding sites. HNF4α promotes *Cyp1a2* expression and is downregulated with age, which could explain the reduction in *Cyp1a2* expression. Overall, our study supports the broad impact of epigenetic aging on xenobiotic metabolism and transport. Future work should evaluate the interplay between hepatic nuclear receptor function and epigenetic aging. These results may have implications for studies of longevity and healthy aging.

**Supplementary Information:**

The online version contains supplementary material available at 10.1007/s11357-024-01137-9.

## Introduction

Older adults aged 65 + are up to seven times more likely to experience adverse drug reactions (ADRs) requiring hospitalization than younger individuals [1]. Physiological changes with age can influence ADR risk, by impairing the body’s ability to safely process drugs [2]. These changes include reduced hepatic blood flow, declining hemostatic reserve, and altered body composition, which can affect drug absorption, distribution, metabolism, and excretion (ADME) processes [3]. However, there is substantial individual variation in the timing and the extent to which these physiological changes occur, and drug response does not correlate well with chronological age. Therefore, a deeper understanding of the age-related physiological changes that affect ADME processes is necessary.

ADME processes involve the action of several classes of drug-metabolizing enzymes and transporters expressed in the liver, kidney, and other organs, with the liver being the primary site of xenobiotic metabolism. Considering the effects of aging, the current consensus is that hepatic clearance of some drugs can be reduced by up to 30% in older adults and phase I reactions mediated by the cytochrome P450 (CYP450) enzymes are more likely to be impaired than phase II (conjugative) metabolism [4]. However, existing studies on the age-related changes to the regulation of specific drug metabolizing enzymes and transporters are inconsistent. For example, many older studies found limited differences with age or changes to just a few specific CYP450 isoforms. On the other hand, more recent studies investigating genome-wide gene expression have shown widespread age-related differential expression of many hepatic genes involved in xenobiotic metabolism and transport, in both humans and mice [5–7]. This suggests that the focus of older studies may have been too narrow and more extensive characterization of xenobiotic gene regulation in the aging liver is warranted.

In recent years, epigenetic changes with age have been recognized as one of the hallmarks of the aging process [8]. Epigenetic processes profoundly affect the expression of genes involved in xenobiotic metabolism and transport [9, 10] and upregulation of genes involved in xenobiotic detoxification is a common mechanism of increased longevity across phyla [11, 12]. Recent studies in laboratory mice suggest that epigenetic aging may substantially alter the hepatic expression and function of some ADME genes [13, 14]. However, comprehensive genome-wide studies of epigenetic aging effects on ADME genes have been limited. Only two studies to date have examined genome-wide epigenetic aging (DNA methylation change) in human liver. Bacalini et al. (2019) [15] studied human post-mortem liver tissue in a limited sample size of brain-dead, heart-beating donors, while Bysani et al. (2017) [16] studied liver biopsies collected during surgery as a component of the Kuopio Obesity Surgery Study. The nature of these samples, being long-term coma patients and obese, respectively, may limit the generalizability of the findings to the normal aging population. Studies in laboratory rodent models of aging have, for example, examined changes to hepatic genome-wide DNA methylation levels in female mice in the context of high fat diet [17]. However, analysis of hepatic epigenetic aging with a focus on its impact on xenobiotic/drug metabolism pathways is lacking. Therefore, in the present study, our goal is to identify age-associated differentially methylated sites (a-DMS) at genes involved in ADME processes in liver tissue from male mice aged at the National Institute of Aging rodent colonies. Due to the large number of ADME genes expressed in the liver, we will first screen the genome for a-DMS using reduced representation bisulfite sequencing (RRBS) [18, 19]. RRBS provides good coverage of CpG islands and regulatory regions [19] at a fraction of the cost of whole genome bisulfite sequencing. Then, for a selection of the most important ADME genes identified in the RRBS screen, we will investigate the relationship between epigenetic aging and expression.

## Methods

### Subjects and DNA extraction

C57BL6 mouse liver tissue samples were obtained from male mice aged 4 and 24 months of age (six mice per age group, in discovery and replication samples) from the National Institute on Aging Rodent tissue bank. Genomic DNA was extracted using the AllPrep DNA/RNA Mini kit (Qiagen). DNA quality and quantity were assessed using a Nanodrop spectrophotometer (ThermoFisher).

### Reduced representation bisulfite sequencing (RRBS)

Six hundred nanograms of genomic DNA per sample were digested using MspI (New England Biolabs) at 37 °C for 16 h and purified using the QIAquick PCR purification kit (Qiagen). Size selection of DNA fragments 100–220 bp from the purified MspI-digested DNA used a Pippin Prep (Sage Science) with a 2% ethidium-free agarose gel cassette. The captured DNA was subjected to bisulfite conversion using the EZ DNA Methylation Gold kit (Zymo Research) according to the manufacturer’s protocol. Bisulfite-converted DNA was used as input for the Accel-NGS Methyl-Seq DNA Library kit (Swift Biosciences, now IDT) to create the RRBS libraries. Library concentrations were measured using a Qubit (ThermoFisher) and size distribution was determined using an Agilent Bioanalyzer. Libraries were sequenced PE150 on an Illumina HiSeq4000 by Azenta/GeneWiz, South Plainfield, NJ.

### RRBS bioinformatics pipeline

FASTQ reads were processed using the Bismark pipeline [20, 21]. First, we used TrimGalore-0.6.6 with the ‘*–rrbs*’ option to eliminate adapter contamination and perform tail trimming, which involved clipping 10 base pairs from the forward and reverse reads, to remove low complexity artifacts of the adaptase library construction method. Read integrity and length were evaluated using FastQC prior to alignment to the (bisulfite-converted) mm10 mouse genome using Bismark-0.23.1. Alignment was performed using the ‘*–non_directional*’ option, which allows for alignment of both the forward and reverse reads of the paired samples. The methylation state of each CpG site was then determined by Bismark methylation extractor with ‘*–no_overlap*’, where overlapping regions in the middle of paired-end reads are called only once.

MethylKit version 1.20.0 was used to quantify and compare methylation levels at each CpG site across samples, to identify age-associated differentially methylated sites (a-DMS). The data were filtered using a minimum coverage of 10 and a high percentage of less than 99.9. Reads were then normalized using the median method and united per age group. Prior to calling a-DMS, we performed Principal Component Analysis (PCA) to identify unmeasured sources of variation in the dataset. Our prior work [22, 23], and that of others [24, 25], has shown this method to be an effective statistical control for unmeasured confounders including cellular heterogeneity in DNA methylation studies. Principal Components (PCs) were tested for association with age, our variable of interest, and those not substantially associated with age were removed in our main analysis. Significant a-DMS were called using false discovery rate (FDR) control [26] at the 5% level.

### Data integration

Top a-DMS from our RRBS analysis in male mouse livers were contrasted with a-DMS from aged female mouse liver samples from Sandoval-Sierra et al. (2020) [17]. Full genome-wide MBD-seq data from this paper were obtained from Dr. Khyobeni Mozhui (personal communication). We identified genes associated with our a-DMS using GREAT (Genomic Regions of Enrichment Annotation Tool) [27] version 4.0.4 (2019) for mm10 with basal plus extension assignment. GREAT was also used for gene ontology/pathway enrichment analysis, using built-in Gene Ontology annotations [28, 29]. Analysis of overlap between liver transcription factor binding sites and RRBS findings used ChIP-Atlas for mm10 [30]. Data for non-liver cell types and liver tumor were excluded. To test for enrichment of HNF4A-regulated genes among genes differentially expressed in aged liver, we used HNFA-regulated genes from ChIP-Atlas (SRX10829257, GEO accession GSM5288563) and expression data from Benayoun et al. (2019) [7]. Liver-expressed genes in adult mice comprised the background set (GXD, Mouse Genome Informatics [31]). Fisher’s exact test was used to obtain *p*-values and odds ratios of overlap, using R Studio running R version 4.2.2.

### Targeted validation of a-DMS using high-resolution melt (HRM) of bisulfite-converted DNA

200 ng of liver genomic DNA was treated with sodium bisulfite using the EZ DNA Methylation kit (Zymo Research, Irvine, CA). Mouse genomic DNA standards of known percentage 5mC (EpigenDx, Hopkinton, MA) were included to create a standard curve. High-resolution melt (HRM) assays were designed using MethPrimer (see Supplementary Table S1 for primer sequences). Assays were run in triplicate using MeltDoctor HRM MasterMix (Applied Biosystems, Foster City, CA) on a Quantstudio 3 instrument. Samples were amplified as follows: 10 min hold at 95 °C followed by 40 cycles of 15 s at 95 °C, 30 s at 57 °C, and 30 s at 72 °C, followed by a final extension at 72 °C for 7 min and a melt curve stage with temperature range of 57 to 95 °C and fluorescence capture at 0.025° per second increment. The net temperature shift (NTS) values of the liver samples were interpolated on the standard curve to yield their 5mC percentage [32].

### Targeted gene expression analysis via quantitative PCR (qPCR)

Targeted gene expression analysis of mouse *Cyp1a2*, *Cyp2d9* and *Abcc2* used pre-designed TaqMan assays from ThermoFisher (Mm00487224_m1, Mm00651731_m1 and Mm00496899_m1 respectively) and standard TaqMan gene expression reagents. *Gapdh* was used as the endogenous control (assay number Mm99999915_g1). Each sample (gene of interest and endogenous control) was run in triplicate on a Quantstudio 3 instrument. The ΔCt method, implemented in the Relative Quantification (RQ) application in the ThermoFisher Cloud, was used to estimate the relative fold change in expression between old (24-month) and young (4-month) age groups.

## Results

### Sequencing statistics, quality control, and global methylation levels

Our sequencing was successful with a mean of 32.9 M sequence read pairs per sample (range 25.1–38.9 M) that aligned well to the mm10 genome within the expected parameters for RRBS (mean 71.1% alignment, range 68.6–76.9%) (Supplementary Table S2). Non-CpG methylation percentages were in the range of 0.4–0.8% showing that the bisulfite conversion worked well. Global methylation of cytosines in a CpG context averaged 34.3% at 4 months versus 33.6% at 24 months, indicating modest genomic hypomethylation with age.

### Genome-wide analysis of age-associated differentially methylated sites (a-DMS)

**Fig. 1:**
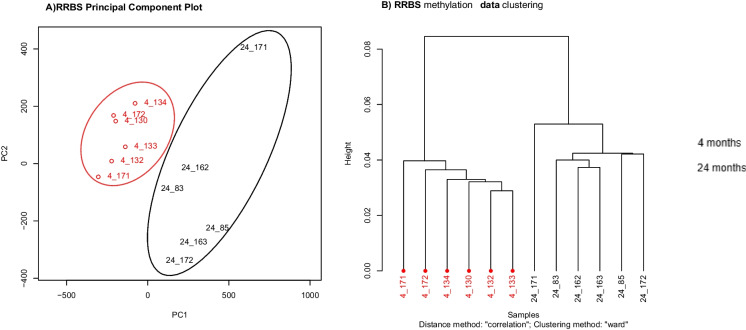
A Plot of sample loadings on the first two principal components of the RRBS DNA methylation data and B Clustering of samples, based on correlation and the Ward clustering method (analysis from MethyKit)

Principal component analysis and clustering by CpG methylation levels showed good separation between individual samples comprising the young and old age groups (Fig. 1). A scree plot of the principal component (PC) loadings showed that the top four PCs accounted for the majority of DNA methylation variation (Supplementary Fig. S1). PC1 and PC2 were associated with age whereas PCs 3 and 4 were not significantly associated with age (Supplementary Table S3). Therefore PCs 3 and 4 were regressed out in our primary analysis.

**Fig. 2:**
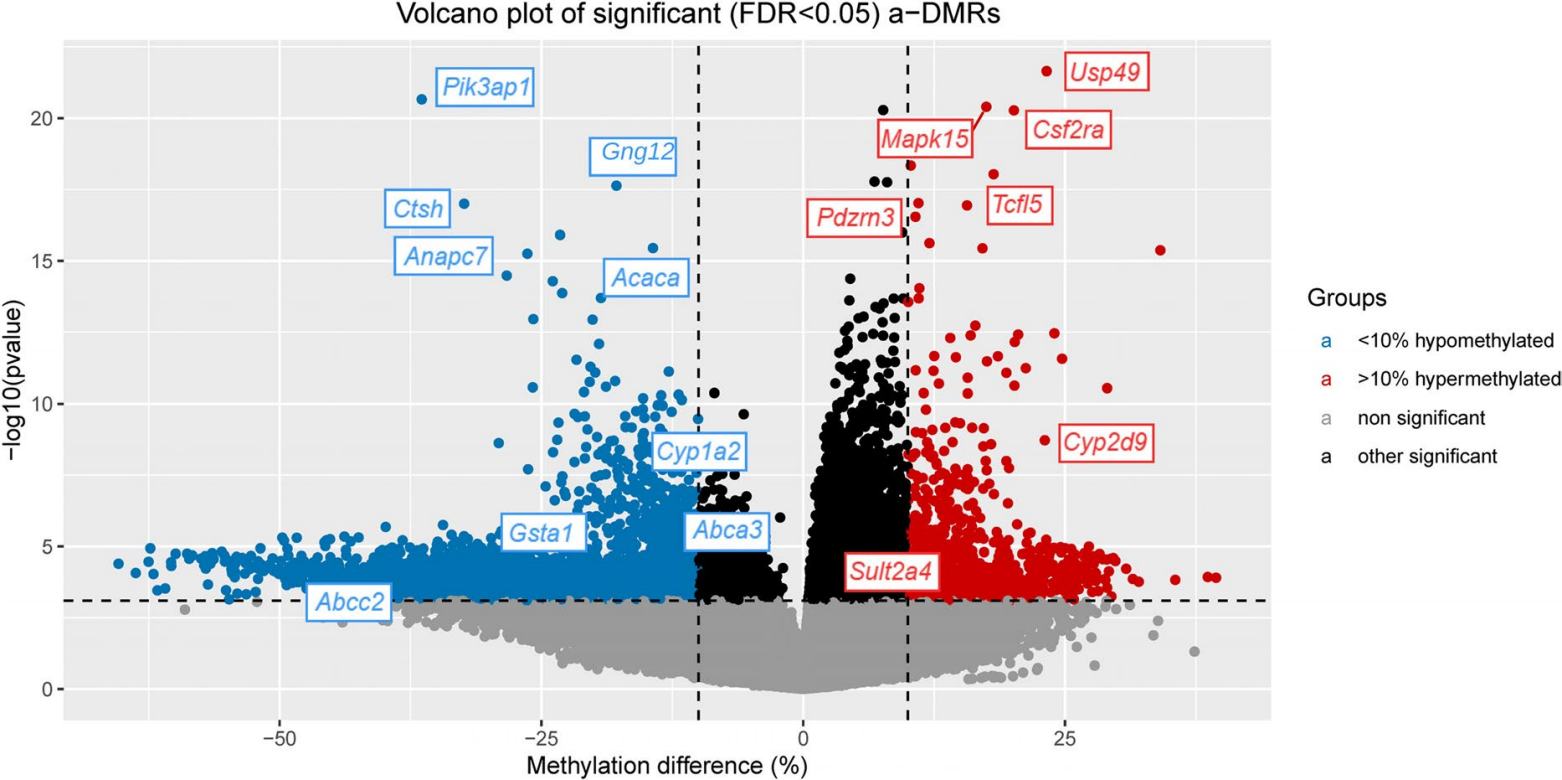
Volcano plot of age-associated differentially methylated sites in mouse liver from age 4 to 24 months. The y-axis is the negative logarithm of the *p*-value, while the x-axis shows the methylation change with age. Genome-wide significance is determined by false discovery rate (FDR) less than 5% (horizontal dotted line). Vertical dotted lines show > 10% hypermethylation and > 10% hypomethylation with age. Genome-wide significant loci showing > 10% hypermethylation with age are color coded red, while > 10% hypomethylation are blue. The genes associated with the top 5 hyper- and hypomethylated loci are shown, as are top xenobiotic metabolism genes and transporters

Analysis of differential methylation with age revealed 12,630 cytosines showing significant (FDR < 0.05) change between the 4- and 24-month age groups. A volcano plot of the findings is shown in Fig. 2 and the complete set of significant results is provided in Supplementary Table S4. Among the significant findings, 7562 (59.9%) were hypermethylated versus 5068 (40.1%) hypomethylated with age. While aging typically causes more hypo- than hypermethylation across the genome, hypermethylation is more prevalent in the CpG-dense regions that are selected by RRBS. This was reflected in the relative positions of the hyper- and hypomethylated sites relative to the transcription start sites (TSS) of genes. Approximately 55% of hypermethylated sites in our data were within 5 kb of a gene TSS, whereas only about 11% of hypomethylated sites were similarly located (Supplementary Figs. S2A-C).

### Integration with a-DMS in female mice

In this study, we ran RRBS on liver DNA from male mice aged 4–24 months. Sandoval-Sierra et al. (2020) [17] previously mapped DNA methylation aging in female mouse livers using a comparable age range of 6–25 months. Although their study was primarily focused on the effects of high fat diet and aging, a-DMS were also reported for the control subjects. We compared our findings with these. Due to the way DNA methylation was analyzed in their study, percentage changes in methylation are not reported. Therefore, we focus on whether nominally (*p* < 0.05) significant changes in the same direction (hyper- vs hypomethylation) were reported by Sandoval-Sierra et al. at the same loci as identified in our analysis. We used exact overlap and a range of distances up to ± 2.5 Kb from our findings. We also examined overlap for all our significant (FDR < 0.05) sites and just those with large effect sizes (≥ 10%) methylation change. Results are shown in Table 1. Broadly speaking, the proportion of overlapping a-DMS in males and females was quite low, not exceeding 20% of sites even when using a ± 2.5 Kb window for overlap for a-DMS showing large (≥ 10%) methylation change. Nevertheless, those sites that did overlap were broadly directionally consistent, with > 90% showing the same effect direction in males and females across all almost all categories analyzed.

**Table 1:** Overlap of age-associated differentially methylated sites (a-DMS) in male mouse liver with published a-DMS in female mouse liver [17]

		exact	± 250 bp	± 1000 bp	± 2500 bp
All a-DMS	Total overlap males/females	367 (3%)	692 (5.5%)	1046 (8.3%)	1509 (11.9%)
	Proportion same direction	93.4%	92.8%	91.5%	85.7%
a-DMS > 10% 5mC change	Total overlap males/females	267 (6.1%)	480 (9.9%)	667 (13.8%)	900 (18.6)
	Proportion same direction	95.5%	94.6%	94.3%	92.3%

### Xenobiotic metabolism genes are implicated by significant a-DMS

**Fig. 3:**
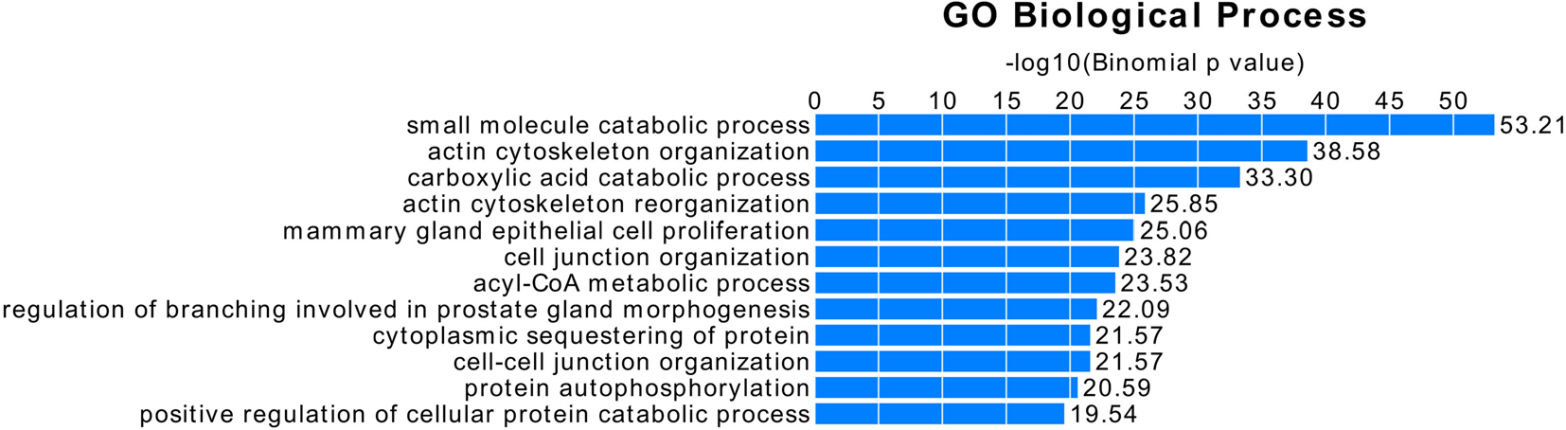
Gene ontology (GO) enrichment analysis of genes with age-associated differentially methylated sites > 10% hyper- or hypomethylated in our primary analysis. The most enriched biological processes are shown ordered by decreasing statistical significance. Analysis and plot from GREAT

We used GREAT [27] to assign genes to our significant (FDR < 0.05) a-DMS based on proximity and regulatory information. The vast majority of both hyper- and hypomethylated sites were associated with one or more genes (Supplementary Figs. S3A-C). The complete set of 12,630 significant sites picked 6,929 (32%) of the 21,395 mouse protein coding genes annotated in GREAT. Focusing on just the 4,836 a-DMS showing larger age-related effects, i.e. ≥ 10% hyper- or hypomethylation (Fig. 2), implicated 3,425 (16%) of all 21,395 genes. The gene ontologies implicated by these genes are shown in Fig. 3. The most significantly enriched pathway is “small molecule catabolic process,” which includes several xenobiotic metabolizing enzymes and transporters. Significant a-DMS (FDR < 0.05) that implicate ADME genes, based on GREAT, are shown in Supplementary Table S5. In this table, we included all genes encoding cytochrome P450 enzymes, Phase II drug metabolism enzymes (sulfotransferases, glutathione S-transferases (GSTs), UDP-glucuronosyltransferases (UGTs)) and the two major classes of transporters, ATP-binding cassette (ABC) transporters and solute carrier (SLC) transporters. For many of these enzymes and transporters the nature of their substrates, whether endogenous, xenobiotic or a combination, are not fully elucidated so we have been inclusive and listed all members of these classes in Supplementary Table S5. Notably, however, among our top findings are mouse orthologs of three of the most clinically important human pharmacogenes, known to act on a large range of xenobiotics: 1) *Cyp1a2* is the mouse ortholog of human *CYP1A2*, which encodes an enzyme accounting for 13% of total CYP content in the human liver and is responsible for metabolizing approximately 9% of prescription drugs [33]. 2) *Cyp2d9* is the mouse ortholog of human *CYP2D6*. The human CYP2D6 enzyme, despite comprising a smaller amount of the total CYP pool in the liver than CYP1A2, is responsible for the metabolism of up to 20% of all prescription drugs [34]. 3) The human ortholog of *Abcc2* encodes the multidrug resistance protein 2 (MRP2) transporter, which is one of the most important hepatic transporters involved in the excretion of a wide variety of xenobiotics, particularly conjugates resulting from Phase II metabolism [35]. All three of these genes were linked to significant a-DMS (FDR < 0.05), showed a ≥ 10% change in DNA methylation with age (Fig. 2) and are known to be expressed in liver. Due to the importance of these genes in xenobiotic metabolism and transport, we proceeded to investigate how epigenetic aging may affect their expression.

### Integration of a-DMS with age-related changes in gene expression

**Fig. 4:**
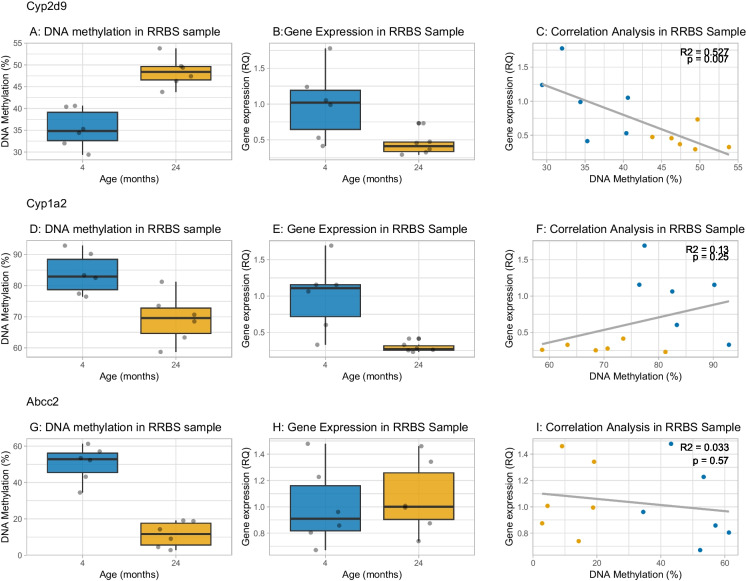
DNA methylation and expression changes with age for *Cyp2d9, Cyp1a2*, and *Abcc2*. Panels **A**), **D**) and **G**) show the most significant change in DNA methylation with age at the three loci, with *Cyp2d9* showing hypermethylation with age, while *Cyp1a2* and *Abcc2* show hypomethylation with age (data from RRBS). Panels **B**), **E**) and H) show the relative quantification of RNA change with age, where 4-month-old samples are set mean = 1.0. Both *Cyp* genes showed lower gene expression with age while there was no significance difference in *Abcc2* expression with age. Panels **C**), **F**) and **I**) show the relationship between methylation levels and expression of the gene. For *Cyp2d9* there was a significant negative relationship between the two variables, whereas for *Cyp1a2* and *Abcc2* there was no significant association between DNA methylation change with age and expression. (*n* = 6 mice per age group)

We extracted RNA from the same biomaterial for which we obtained RRBS data and used qPCR to assay the expression levels of *Cyp1a2*, *Cyp2d9*, and *Abcc2*. The results are shown in Fig. 4, where we show the DNA methylation levels from RRBS at the most significant a-DMS at each gene, in addition to the expression change with age from qPCR and the correlation between DNA methylation and expression. For *Cyp2d9*, there was a 12.5% increase in DNA methylation (*p* = 2.49 × 10^–7^, *q* = 2.3 × 10^–4^). This was accompanied by a significant (*p* = 0.029) decrease in relative expression of *Cyp2d9*, whereby expression at 24 months was 43.3% of that at 4 months. The DNA methylation and expression levels were significantly negatively associated (*p* = 0.007, *R*^2^ = 0.53). This indicated that the age-associated DNA hypermethylation at *Cyp2d9* was associated with lower gene expression, which is in the expected direction because DNA methylation is typically repressive of expression. At the *Abcc2* a-DMR, the very large (36.4%) decrease in DNA methylation with age was not accompanied by any change in expression with age. *Cyp1a2* showed a 15.7% decrease from 4 to 24 months (*p* = 7.56 × 10^–8^, *q* = 9.89 × 10^–5^). This hypomethylation was accompanied by a significant (*p* = 0.005) *decrease* in relative expression of *Cyp1a2*, whereby expression at 24 months was 29.4% of that at 4 months. The DNA methylation levels and expression levels were not significantly associated (*p* = 0.527). Therefore, at *Cyp1a2*, the substantial hypomethylation with age was not driving the observed reduction in gene expression. Since methylation is typically repressive of expression, this finding could be viewed as being in the opposite direction to expectations.

### Validation and replication of findings

We examined the data from Sandoval-Sierra et al. (2020), as described above, to see if similar DNA methylation effects were observed in females for the a-DMS at *Cyp1a2*, *Cyp2d9* and *Abcc2*. While their assay did not cover the a-DMS at *Abcc2*, overlapping positions were analyzed for our a-DMS at *Cyp2d9* and *Cyp1a2*. However, no significant age-related changes in DNA methylation were found at either of these sites in females, suggesting that any change is likely specific to males only. To assess the robustness of our gene expression findings, we checked them against the results of an RNA-seq analysis of aging mouse liver by Benayoun et al. (2019) [7]. This study used male mice of comparable ages (3–29 months). Their expression findings were in broad agreement with ours, whereby they also found *Cyp2d9* (*p* = 1.11 × 10^–4^, *q* = 0.007) and *Cyp1a2* (*p* = 2.68 × 10^–4^, *q* = 0.013) to be significantly down-regulated in older subjects. Benayoun et al. also reported no significant change in *Abcc2* expression, in agreement with our study. Therefore, we did not consider this gene further.

**Fig. 5:**
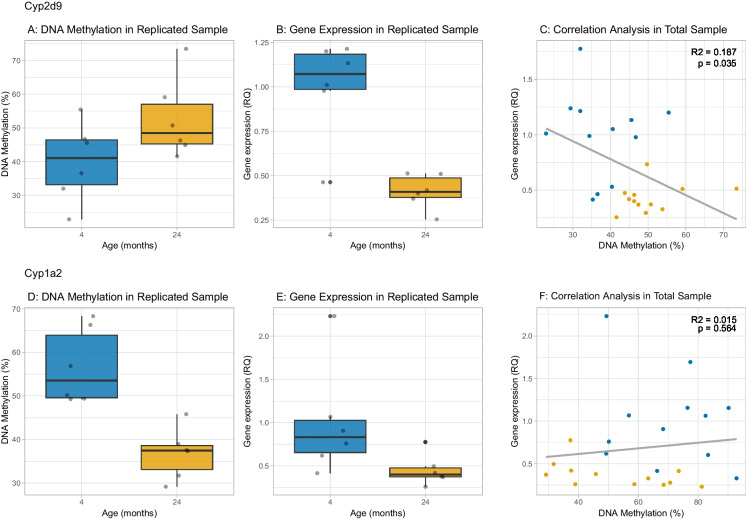
Replication of DNA methylation and expression changes with age for *Cyp2d9* and *Cyp1a2*. Panels **A**) and **D**) show the change in DNA methylation with age at the most significant a-DMS at *Cyp2d9* and *Cyp1a2* respectively, in an independent sample of male subjects (*n* = 6 for each age group of 4 and 24 months old) using a targeted technology, high resolution melt (HRM) analysis of bisulfite converted DNA. Panels **B**) and E show the change in gene expression with age for *Cyp2d9* and *Cyp1a2* respectively, in the replication sample. Panel **C**) shows a significant negative relationship between *Cyp2d9* DNA methylation and expression, whereby increased DNA methylation leads to reduced expression, in the combined discovery and replication samples (*n* = 12 for each age group of 4 and 24 months old). Panel **F**) shows the lack of relationship between methylation and expression of *Cyp1a2* in the combined discovery and replication samples

To replicate our DNA methylation and gene expression findings with the two CYP genes, we proceeded to analyze them in an independent sample of livers from male mice (also *n* = 6 per age group, 4 and 24 months). We assayed DNA methylation at the significant sites from RRBS using a different, targeted technology i.e. high-resolution melt (HRM) analysis of bisulfite-converted DNA. We replicated the most significant a-DMS and the change in expression for both genes. Thus, for *Cyp2d9*, we saw a 12.8% increase from 4 to 24 months (*p* = 0.044, Fig. 5A), while for *Cyp1a2* we saw a 19.96% decrease (*p* = 0.001, Fig. 5D). We also observed a significant decrease in *Cyp2d9* expression in the replication sample whereby expression at 24 months was 41% of that at 4 months (*p* = 0.001 Fig. 5B), while *Cyp1a2* showed a significant reduction to 45% at 24 months (*p* = 0.046, Fig. 5E). For *Cyp2d9*, the negative association between DNA methylation and gene expression persisted in the combined discovery and replication samples (*p* = 0.035, *R*^2^ = 0.187, Fig. 5C). However, for *Cyp1a2* there was no association between DNA methylation change with age and expression (Fig. 5F). Therefore, we concluded that the local age-related hypomethylation at *Cyp1a2* is not driving the observed change in gene expression. For our next steps, we sought to identify alternative mechanisms that could explain the reduction in *Cyp1a2* expression with age.

### Distal effects of DNA methylation aging on expression of Cyp1a2

We hypothesized that distal (long-range) hypermethylated a-DMS could be influencing the reduced *Cyp1a2* expression. Therefore, we analyzed the association between *Cyp1a2* expression and the methylation levels of all a-DMS in our data across the entire genome. None yielded sufficiently strong association with *Cyp1a2* expression to pass correction for multiple testing (FDR < 0.05). This largely ruled out the possibility of a long-range a-DMS being a significant cause of the reduction in gene expression at *Cyp1a2* with age, at least for any sites that are captured in our RRBS data.

### Locus overlap analysis with hypomethylated a-DMS

We hypothesized that changes to the function of relevant transcription factors with age could lead to reduced expression, even if a gene was undergoing hypomethylation. To identify candidate transcription factors, we tested for enrichment of hepatic transcription factor binding sites at our hypomethylated a-DMS. We used publicly available ChIP-seq data in mouse liver and the results are shown in Table 2. Eight out of the top 10 overlapping ChIP-seq profiles were for hepatocyte nuclear factor 4 alpha (HNF4α), which was very significantly enriched at our hypomethylated a-DMS. By comparison, hypermethylated a-DMS mostly showed enrichment for CTCF (CCCTC-binding factor), a transcriptional regulator protein known to be associated with epigenetic aging sites [36], as shown in Supplementary Table S6.

**Table 2:** Enrichment of hepatic transcription factor binding sites at hypomethylated a-DMS

ChIP- Atlas ID	GEO ID	TF	Cell	N peaks	Over lap	Exp	-log pval	-log qval	Fold Enrich
SRX10829257	GSM5288563	HNF4A	Liver	56,351	744	58	162.9	159.7	12.83
SRX10829258	GSM5288564	HNF4A	Liver	43,330	662	45	149.7	146.9	14.71
SRX10829256	GSM5288562	HNF4A	Liver	41,138	591	41	132.1	129.4	14.41
SRX204712	GSM1037670	CEBPB	Liver	42,990	506	39	108.9	106.3	12.97
SRX20201040	GSM7290595	HNF4A	Liver	58,639	531	64	96.8	94.3	8.30
SRX20201038	GSM7290593	HNF4A	Liver	54,944	488	60	87.8	85.4	8.13
SRX10829260	GSM5288566	HNF4A	Liver	25,015	374	25	83.0	80.7	14.96
SRX20201039	GSM7290594	HNF4A	Liver	55,716	452	59	78.8	76.6	7.66
DRX178625	GSM7290593	NFIL3	Liver	22,912	351	23	78.2	76.0	15.26
SRX11141649	GSM5381235	HNF4A	Liver	42,777	442	56	78.1	76.0	7.89

We inspected the top HNF4α ChIP-seq track (GSM5288563 in Table 2), which showed evidence for HNF4α binding at the *Cyp1a2* hypomethylated a-DMR, but not at *Abcc2* (Supplementary Fig. S4). Notably, reduced expression with age was observed at *Cyp1a2* but not *Abcc2* in our data. As a more general analysis, we tested if HNF4α-regulated genes from ChIP-Atlas were enriched among liver genes differentially expressed with age [7]. We found that HNF4α genes were more likely to be downregulated with age (*p* = 0.042, OR = 2.04) but not upregulated with age (*p* = 0.966, OR = 0.72). These findings further support the potential role of HNF4α in the downregulation of hepatic genes in aging.

## Discussion

In this paper, we show that epigenetic aging affects a substantial number of genes involved in xenobiotic metabolism and transport. Our extensive mapping of a-DMS is consistent with the broad extent of DNA methylation changes observed with age in other tissues, both in model organisms and humans. Notably, mouse genes encoding orthologs of important human cytochrome P450 enzymes and the multidrug resistance protein 2 (MRP2) transporter were found to undergo epigenetic aging. However, the relationship between DNA methylation aging and gene expression is complex. The hypermethylated a-DMS at *Cyp2d9* (chr15:82,494,252) was located in an ENCODE candidate cis-regulatory element (cCRE) > 30 kb from the gene, with the functional linkage between the two initially assigned by GREAT [27] and supported by our data, whereby DNA methylation at this position was associated with reduced expression of that gene in aging. The most significant a-DMS at *Abcc2* (chr19:43,886,324) involved hypomethylation at an ENCODE cCRE (distal enhancer) approximately 35 kb from the gene, as assigned by GREAT, but no change in *Abcc2* hepatic expression with age was found in our study or that of Benayoun et al. [7]. The most significant a-DMS at *Cyp1a2* (chr9:57,681,974) involved hypomethylation at exon 6 of the gene. While we might expect local hypomethylation to be associated with an increase in expression due to opening of chromatin, there was no association between methylation at this position and expression. In fact, the observed reduction in gene expression at *Cyp1a2* is opposite to the effect direction we might expect from hypomethylation. We hypothesized that additional *trans*-acting factors are at play, whereby reduced levels of specific transcription factors (TFs) may be more important than local DNA methylation in the expression of some hepatic genes in aging. Indeed, hypomethylated liver a-DMS in our study showed significant enrichment of hepatocyte nuclear factor 4-alpha (HNF4α) binding sites. This hepatic TF belongs to the nuclear receptor superfamily and is involved in regulating a wide range of critical biological processes [37]. In the liver, it is master regulator of hepatocyte differentiation and a key determinant for liver function [38]. Furthermore, it is substantially downregulated in aged mice [39], rats [40] and humans [41], and is an activator of *CYP1A2*. Taken together, these observations suggest that loss of this TF could explain reduced *Cyp1a2* expression in aging.

Limitations of the study include that 1) RRBS does not capture the entire methylome, so we will inevitably miss some epigenetic aging effects, 2) bisulfite-based methods as used here cannot distinguish between 5-methylcytosine and other marks at the same position, e.g., 5-hydroxymethylcytosine, so future work should focus on parsing the relative proportions of such marks at the a-DMS that we identified, 3) our RRBS was in males only, although we did compare our results to prior published analyses in females and the sex-dependent variation we observed is consistent with prior studies [42], 4) we only analyzed expression at a selection of relevant genes, rather than conducting genome-wide expression analysis and 5) putative relationships identified between expression and DNA methylation aging effects are associative only and that true causality may only be determined by further work that directly manipulates epigenetic states. Despite these limitations, the results of our study have potential implications, as we now outline.

Our study revealed a-DMS at several genes encoding cytochrome P450s, Phase II xenobiotic metabolism enzymes, ABC transporters and SLC transporters. Indeed, we identified epigenetic aging effects at the mouse orthologs of some of the most important human drug metabolizing genes and transporters: *Cyp1a2, Cyp2d9,* and *Abcc2* [33–35]. While the epigenetic changes at *Abcc2* were not accompanied by a change in expression, the expression levels of both CYPs were downregulated with aging. A large body of research in laboratory animals has demonstrated substantial age-related declines in CYP content, activity, and inducibility [40, 43]. In humans, some reduction in Phase I (CYP-mediated) metabolism is considered likely, although Phase II metabolism is generally considered to be intact in older adults. In a highly cited 2009 review, Klotz [4] concluded that hepatic drug clearance can be reduced by up to 30% in older adults. A more recent study by Dücker and Brockmöller (2019) [44] reviewed pharmacokinetic data in young and old patients and upheld the idea that drug metabolism and transport processes are somewhat impaired in older adults. However, studies involving pharmacokinetic analysis of enzyme and transporter activity, rather than gene or protein expression, are more challenging to interpret in the context of epigenetic aging. When based on drug metabolism rates in living humans, measurements of drug metabolism could be affected by issues such as reduced liver mass and blood flow that are known to affect older adults and any reduction in activity may not be directly related to reduced expression.

Considering studies that measured liver enzyme and transporter expression, there are conflicting findings. George et al. [45] analyzed the microsome content of human liver tissues and found that the total hepatic cytochrome P450 enzyme levels declined by (-3.5%) for each decade of life. Sotaniemi et al. [46] analyzed 226 human subjects and reported a 32% reduction in cytochrome P450 content and a 29% reduction in activity in patients 70 years and older when compared to patients aged 20–29 years. On the other hand, Yang et al. [47] looked at hepatic gene expression networks in a large sample of approximately 400 subjects and concluded that cytochrome P450 gene expression increases with age, contradicting prior research.

Overall, existing evidence favors reduced hepatic CYP activity in aged rodents, with some evidence to suggest that some reduction is present in humans. The mechanisms through which this can occur are likely to be manifold. While epigenetic aging may affect chromatin state, we have shown that DNA methylation is an imperfect proxy of gene expression. We found some evidence that HNF4α may be involved in the reduced gene expression with age for some loci, including *Cyp1a2*. Future studies could look to evaluating HNF4α binding patterns and activity in the aging liver, in addition to mapping other epigenetic marks such as histone modifications. It should also be noted that other molecular mechanisms have been postulated as affecting gene expression in the aging liver. Polymerase pausing is one such mechanism, where RNA polymerase fails to work as it should even in open chromatin positions, leading to reduced gene expression [48, 49].

Further understanding of these mechanisms could have clinical implications. First, if we can identify the mechanisms underlying compromised xenobiotic metabolism, this could result in the development of better biomarkers to modulate drug dosing in older adults. Moreover, “intact” xenobiotic metabolism, which has not declined in old age, is strongly associated with increased longevity. Mutation of xenobiotic metabolizing genes in several organisms can affect lifespan [50–53] and genetic variation in xenobiotic metabolizing genes has been associated with longevity in several human studies [54–56]. There is now substantial and convincing evidence from worm to mouse to human that upregulation of genes involved in xenobiotic detoxification is a common mechanism of increased longevity across phyla [11, 12]. Therefore, a deeper understanding of mechanisms governing expression of xenobiotic metabolism genes could increase our understanding of the biology of healthy aging. Future studies could apply single cell sequencing methods, to obtain a more refined perspective of these mechanisms in specific cell types.

## Supplementary Information

Below is the link to the electronic supplementary material.

Supplementary file1 (DOCX 187 KB)

Supplementary file2 (XLSX 860 KB)

Supplementary file3 (XLSX 52 KB)

## Data Availability

RRBS raw sequence reads have been submitted to the National Center for Biotechnology Information (NCBI) sequence read archive under Bioproject accession number PRJNA1089703 (www.ncbi.nlm.nih.gov/bioproject/PRJNA1089703).
