# Supplementary material for: Genome-wide analysis of hepatic DNA methylation reveals impact of epigenetic aging on xenobiotic metabolism and transport genes in an aged mouse model
Source: GeroScience. 2024 Apr 1;46(6):5967–80. doi: 10.1007/s11357-024-01137-9 (PMC11493898; doi:10.1007/s11357-024-01137-9)
Supplement: Supplementary file 1 — Supplementary file1 (DOCX 187 KB) [file 11357_2024_1137_MOESM1_ESM.docx]

Title: Genome-wide analysis of hepatic DNA methylation reveals impact of epigenetic aging on xenobiotic metabolism and transport genes in an aged mouse model

Sara Abudahab ^a^, Mohamad M. Kronfol ^a^, Mikhail G. Dozmorov ^b,c^, Thomas Campbell ^d^, Fay M. Jahr ^a^, Jasmine Nguyen ^a^, Ola AlAzzeh ^a^, Dalia Y. Al Saeedy ^a^, Ashley Victor ^a^, Sera Lee ^a^, Shravani Malay, Dana Lapato ^e^, Matthew Halquist ^d^, MaryPeace McRae ^a^, Laxmikant S. Deshpande ^f,g^, Patricia W. Slattum ^a^, Elvin T. Price ^a^, Joseph L. McClay ^a*^

^a^ Department of Pharmacotherapy and Outcomes Science, School of Pharmacy, Virginia Commonwealth University, Richmond, Virginia, USA.

^b^ Department of Biostatistics, Virginia Commonwealth University, Richmond, Virginia, USA.

^c^ Department of Pathology, Virginia Commonwealth University, Richmond, VA, USA.

^d^ Department of Pharmaceutics, Virginia Commonwealth University, Richmond, VA, USA.

^e^ Department of Human Genetics, Virginia Commonwealth University, Richmond, VA, USA.

^f^ Department of Neurology, Virginia Commonwealth University, Richmond, VA, USA.

^g^ Department of Pharmacology and Toxicology, Virginia Commonwealth University, Richmond, VA, USA.

**Contents**

**Table S1**: Primer sequences p3

**Table S2**: Sequencing statistics. P3

**Figure S1**: Scree plot of principal components p4

**Table S3**: Principal component associations with age p4

**Table S4**: All significant a-DMRs (FDR<0.05) Excel file

**Figure S2**: **A)** Histogram of distance to closest TSS for all a-DMRs p5

**B)** Hypermethylated a-DMRs distance to closest TSS p5

**C)** Hypomethylated a-DMRs distance to closest TSS p5

**Figure S3**: **A)** Histogram of number of genes per a-DMR assigned by GREAT p6

**B)** Numbers of genes associated with hypermethylated a-DMRs p6

**C)** Numbers of genes associated with hypomethylated a-DMRs p6

**Table S5**: ADME genes with significant a-DMRs Excel file

**Figure S4**: HNF4α binding at two ADME genes p7

**Table S6:** Transcription factor binding site enrichment at hypermethylated a-DMRs p8

**Table S1. Sequence of primers used for targeted high resolution melt DNA methylation analysis**

| Target | Forward | Reverse |
| --- | --- | --- |
| *Cyp1a2* | *5’GATGTTTGTTGTTGATGTTTAGGTAAA’3* | *5’ATAAAAACACAAACCCCTTTCAAT’3* |
|  |  |  |
| *Cyp2d9* | *5’GTTGATGTTTAGGTAAAGTATTTTTG’3* | *5’TAAAACACAACATCTACTCCAATCC’3* |
|  |  |  |

**Table S2. RRBS read mapping statistics**

| Sample ID | Sequence pairs analyzed in total | Number of paired-end alignments with a unique best hit | Mapping efficiency | Total number of C's analyzed | C methylated in CpG context | Total methylated C's in CHG context | Total methylated C's in CHH context |
| --- | --- | --- | --- | --- | --- | --- | --- |
| 4-130 | 34400495 | 23599547 | 68.60% | 1470197753 | 49512454 (35%) | 1965779 (0.6%) | 6468520 (0.7%) |
| 4-132 | 35239705 | 24786859 | 70.30% | 1582657663 | 58632110 (35.8%) | 2854409 (0.8%) | 8760547 (0.8%) |
| 4-133 | 38942221 | 27071575 | 69.50% | 1724630394 | 63042938 (34.1%) | 1869125 (0.5%) | 6408915 (0.6%) |
| 4-134 | 31813476 | 22270902 | 70.00% | 1395814339 | 47259415 (33.1%) | 1975297 (0.6%) | 6932421 (0.8%) |
| 4-171 | 37757311 | 26094679 | 69.10% | 1650070263 | 64312873 (36.5%) | 2031663 (0.5%) | 7092423 (0.7%) |
| 4-172 | 31165405 | 22249413 | 71.40% | 1414284970 | 47058525 (31%) | 1468675 (0.4%) | 5013361 (0.5%) |
|  |  |  |  |  |  |  |  |
| 24-83 | 29348399 | 20483428 | 69.80% | 1304554830 | 45292261 (33.6%) | 1802648 (0.6%) | 5301568 (0.6%) |
| 24-85 | 35721548 | 25938185 | 72.60% | 1641726777 | 53588182 (32.4%) | 2658387 (0.7%) | 7789433 (0.7%) |
| 24-162 | 30491907 | 21320879 | 69.90% | 1375801419 | 47775556 (30.5%) | 1657328 (0.5%) | 5372836 (0.6%) |
| 24-163 | 32270195 | 23375793 | 72.40% | 1477080602 | 54100230 (34.8%) | 1838400 (0.5%) | 5763666 (0.6%) |
| 24-171 | 32755997 | 24904415 | 76.00% | 1577948859 | 52457805 (34.3%) | 1585438 (0.4%) | 4761875 (0.5%) |
| 24-172 | 25117205 | 18395907 | 73.20% | 1175357546 | 43241591 (35.8%) | 1827567 (0.7%) | 5538138 (0.7%) |

**Figure S1. Scree plot of the top principal components obtained from PCA of RRBS data**


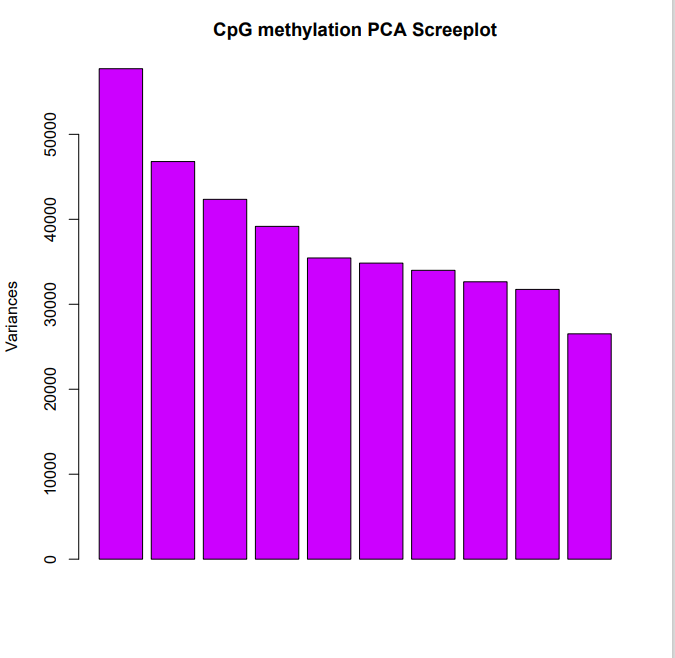


**Table S3. PCs Associations with age**

| PC | p-value |
| --- | --- |
| PC1 | 0.052 |
| PC2 | 7.10 x10^6^ |
| PC3 | 0.313 |
| PC4 | 0.946 |
| PC5 | 0.807 |
| PC6 | 0.856 |
| PC7 | 0.996 |
| PC8 | 0.922 |
| PC9 | 0.972 |
| PC10 | 0.989 |
| PC11 | 0.821 |
| PC12 | 0.839 |

**Figure S2A – All a-DMRs Distance to TSS**


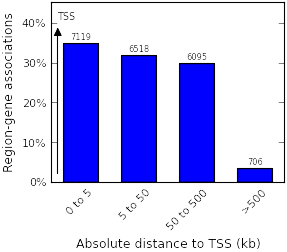


**Figure S2B – Hypermethylated a-DMRs Distance to TSS**


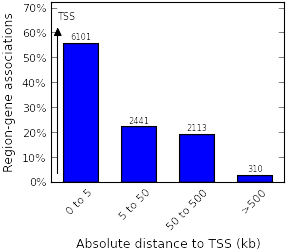


**Figure S2C – Hypomethylated a-DMRs Distance to TSS**


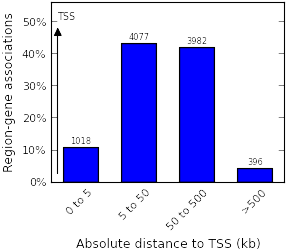


**Figure S3All a-DMRs - number of genes assigned by GREAT**


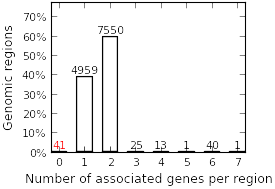


**Figure S3B Hypermethylated a-DMRs number of genes assigned by GREAT**


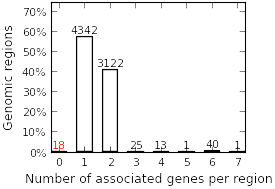


**Figure S3C Hypomethylated a-DMRs number of genes assigned by GREAT**


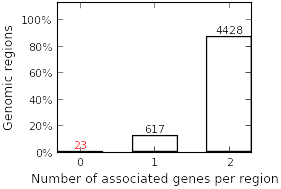


**Figure S4.** HNF4α binding: Upper panel *Abcc2*, Lower panel *Cyp1a2*

**
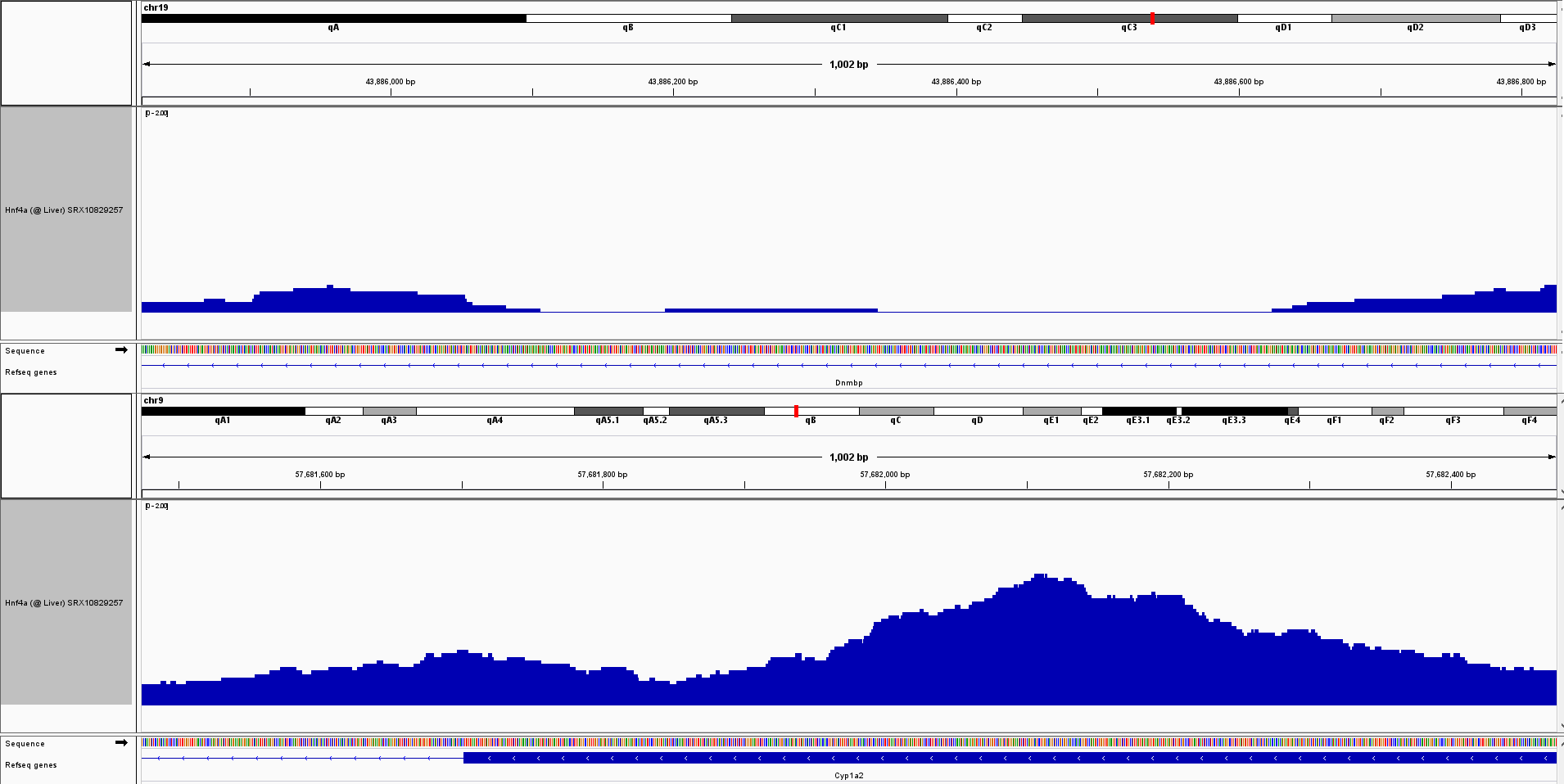
**

Integrative Genomics Viewer (IGV) plots around our a-DMRs associated with *Abcc2* (chr19:43886324, upper panel, intron of *Dnmbp*) and *Cyp1a2* (chr9:57681974, lower panel) for the HNF4A ChIP-seq track most enriched with our hypomethylated binding sites (ChIP-Atlas accession SRX10829257, GEO accession GSM5288563: BWT HNF4A ZT16 ChIPseq 2; Mus musculus; ChIP-Seq). Gene assignments were based on GREAT.

**Table S6. Transcription factor binding site enrichment at hypermethylated a-DMRs (FDR<0.05)**

| ID | Antigen class | Antigen | Cell | Num of peaks | Overlaps / dataset A | Log P-val | Log Q-val | Fold Enrichment |
| --- | --- | --- | --- | --- | --- | --- | --- | --- |
| SRX9250896 | TFs and others | Ctcf | Liver | 30590 | 661/7562 | -158.0 | -155.5 | 20.03 |
| SRX9250886 | TFs and others | Ctcf | Liver | 25938 | 513/7562 | -121.3 | -118.9 | 19.73 |
| SRX6924491 | TFs and others | Tead1 | Liver | 37069 | 588/7562 | -115.6 | -113.4 | 10.32 |
| SRX9250894 | TFs and others | Ctcf | Liver | 24920 | 483/7562 | -114.5 | -112.4 | 20.12 |
| SRX16061857 | TFs and others | Ctcf | Liver | 32947 | 462/7562 | -107.4 | -105.2 | 18.48 |
| SRX3120282 | TFs and others | Ctcf | Liver | 16417 | 410/7562 | -106.3 | -104.2 | 34.17 |
| SRX9250884 | TFs and others | Ctcf | Liver | 25004 | 458/7562 | -106.2 | -104.2 | 18.32 |
| SRX1486519 | TFs and others | Myc | Liver | 7734 | 389/7562 | -105.2 | -103.2 | 48.63 |
| SRX9250898 | TFs and others | Ctcf | Liver | 22876 | 421/7562 | -98.4 | -96.5 | 19.14 |
| SRX9250890 | TFs and others | Ctcf | Liver | 22844 | 419/7562 | -97.9 | -96.0 | 19.05 |

Data from ChIP-Atlas (<https://chip-atlas.org/>) for mm10, selection of TFs in liver. Analysis with permutation x100. Non-standard liver cells and tumor cells were excluded.
